# Protein secretion and outer membrane assembly in *Alphaproteobacteria*

**DOI:** 10.1111/j.1574-6976.2008.00130.x

**Published:** 2008-08-28

**Authors:** Xenia Gatsos, Andrew J Perry, Khatira Anwari, Pavel Dolezal, P Peter Wolynec, Vladimir A Likić, Anthony W Purcell, Susan K Buchanan, Trevor Lithgow

**Affiliations:** 1 Department of Biochemistry and Molecular Biology, University of MelbourneMelbourne, Australia; 2 Bio21 Molecular Science and Biotechnology Institute, University of MelbourneMelbourne, Australia; 3 Laboratory of Molecular Biology, National Institute of Diabetes and Digestive and Kidney Diseases, National Institutes of HealthBethesda, MD, USA

**Keywords:** outer membrane assembly, membrane structure, Omp85, β-barrel proteins, *Alphaproteobacteria*, mitochondria

## Abstract

The assembly of β-barrel proteins into membranes is a fundamental process that is essential in Gram-negative bacteria, mitochondria and plastids. Our understanding of the mechanism of β-barrel assembly is progressing from studies carried out in *Escherichia coli* and *Neisseria meningitidis*. Comparative sequence analysis suggests that while many components mediating β-barrel protein assembly are conserved in all groups of bacteria with outer membranes, some components are notably absent. The *Alphaproteobacteria* in particular seem prone to gene loss and show the presence or absence of specific components mediating the assembly of β-barrels: some components of the pathway appear to be missing from whole groups of bacteria (e.g. Skp, YfgL and NlpB), other proteins are conserved but are missing characteristic domains (e.g. SurA). This comparative analysis is also revealing important structural signatures that are vague unless multiple members from a protein family are considered as a group (e.g. tetratricopeptide repeat (TPR) motifs in YfiO, β-propeller signatures in YfgL). Given that the process of the β-barrel assembly is conserved, analysis of outer membrane biogenesis in *Alphaproteobacteria*, the bacterial group that gave rise to mitochondria, also promises insight into the assembly of β-barrel proteins in eukaryotes.

## Introduction

Bacterial cells precisely organize their cellular activities within the cytoplasmic compartment, and the stability and integrity of a bacterial cell relies on the presence of a cell wall that encases the cytoplasm. The cell wall comprises the cytoplasmic membrane, peptidoglycan layer and, in Gram-negative bacteria, an outer membrane composed of integral membrane proteins, lipids, and lipopolysaccharides (Nikaido, 2003; Scheffers & Pinho, 2005; Ruiz *et al.*, 2006; Bos *et al.*, 2007). Understanding the properties of this structural and biochemical barrier is critical to devising strategies to inhibit growth of bacterial pathogens.

Analysing the structure of membranes and membrane proteins remains a challenging problem, given the difficulty associated with purifying membrane fractions and analysing membrane protein structures (Elofsson & von Heijne, 2007). Yet understanding how membranes function, and particularly how membranes are assembled, depends on knowledge of the structures of the functionally important membrane proteins. As detailed in this review, bacterial outer membranes are largely composed of proteins with a ‘β-barrel’ architecture. Recent research provides the means to appreciate the secretion, folding and assembly of β-barrel proteins in bacterial outer membranes. These recent studies build on classic studies of protein secretion in bacteria, of membrane protein crystallization and biophysical analyses. With the knowledge that mitochondria share an evolutionary relationship with *Alphaproteobacteria*, comparisons between the outer membrane assembly machinery found in mitochondria with that found in bacteria are shedding light on the fundamental aspects of β-barrel protein assembly. Analysis of genome sequences suggests the outer membrane assembly machinery might be similar in all classes of bacteria that have outer membranes.

## Bacterial outer membranes: structure and function

Generally, bacterial outer membranes have been found to be asymmetric, with the inner leaflet of the outer membrane composed mainly of phospholipids and the outer leaflet of lipopolysaccharide. The lipid A moiety of lipopolysaccharide sits within the hydrophobic interior of the membrane and to this is anchored the hydrophilic core polysaccharide, followed by the highly variable O-antigen (Raetz & Whitfield, 2002). Some structural variations on this general theme exist: lipopolysaccharide is sometimes absent (e.g. in *Deinococcus radiodurans*, the spirochaetes *Treponema* and *Borrelia* and *Alphaproteobacteria* such as *Sphingomonas*; Work & Griffiths, 1968; Thompson & Murray, 1981; Hardy & Levin, 1983; Belisle *et al.*, 1994; Kawasaki *et al.*, 1994; Huang & Anderson, 1995; Wiese & Seydel, 1999; Porcella & Schwan, 2001; Raetz & Whitfield, 2002; Cullen *et al.*, 2004), and the peptidoglycan layer can be more intimately associated with the inner, rather than the outer membrane (e.g. in spirochaetes; Holt, 1978). For all these bacteria, a common theme is the presence of a diverse array of β-barrel proteins in the outer membrane (Buchanan, 1999; Schulz, 2000, 2002; Wimley, 2003). There are two other topologies in which outer membrane proteins might be found: lipoproteins (reviewed by Narita *et al.*, 2004; Tokuda & Matsuyama, 2004) anchored to the inner or outer leaflet of the outer membrane by covalently attached lipids, and a new class of proteins typified by Wza, that have an α-/β-structure that has not been observed previously in any type of integral membrane protein (Collins & Derrick, 2007).

**Fig. 1:**
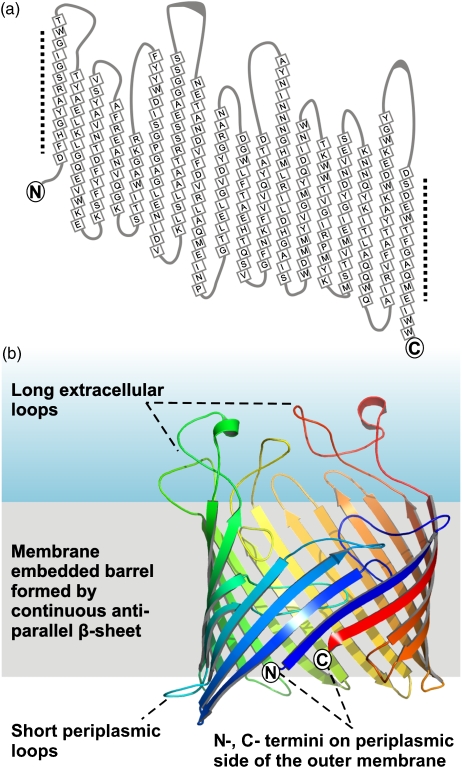
What is a β-barrel? (a) The polypeptide backbone of LamB from *Escherichia coli* (Wang *et al.*, 1997) is traced to show the residues contributing to each β-strand (in squares) aligned to based on the hydrogen bond interactions between each strand. The unpaired hydrogen bond donors and acceptors in the first and last strand are highlighted by the dotted lines. (b) The structure of the assembled β-barrel membrane protein, represented as if in three dimensions, shows sequential β-strands (shown as colored arrows) form an antiparallel sheet that wraps into a cylinder: the final β-strand hydrogen bonds to the first strand to complete the barrel. Loops of polypeptide between the strands tend to be short on the periplasmic rim of the barrel, while longer loops are exposed to the extracellular face of the membrane. These longer loops are structured and can be folded back into the barrel lumen.

The best-characterized class of outer membrane proteins are the β-barrels. In this well-defined structure, the polypeptide chain folds into a series of antiparallel β-strands that join through hydrogen bonds between the first and last β-strand, to form a barrel (Fig. 1a). In cases such as LamB, the N-terminus is exposed to the periplasm (Fig. 1b), while in some β-barrel structures the N-terminus is folded back into the barrel lumen. The outer surface of the β-barrel is formed from amino acid residues with hydrophobic side chains, and the central space of the barrel filled by more hydrophilic (including charged) residues. If the diameter of the barrel is large enough, it will form a water-filled cavity. The cavity can form a pore through the outer membrane, but can be partially (or fully) occluded by the infolding of interstrand loops and the N-terminus (Cowan *et al.*, 1992; Buchanan, 1999; Basle *et al.*, 2006). This provides several possibilities to regulate the semi-permeable nature of the outer membrane, such that β-barrel membrane proteins can define the barrier characteristics of the cell wall in Gram-negative bacteria.

## The *Alpha*- (and other) *proteobacteria*

The structures of almost all known β-barrel proteins are from species of *Proteobacteria*. The *Proteobacteria* are classified into five distinct groups: the *Alpha*-, *Beta*-, *Gamma*-, *Delta*- and *Epsilonproteobacteria* based on molecular phylogenetics of rRNA sequences (Woese, 1987). A number of prediction methods have been developed to detect β-barrel proteins from genome sequence (Wimley, 2002; Berven *et al.*, 2004; Bigelow *et al.*, 2004) and these suggest that most classes of *Proteobacteria* code for more than 100 β-barrel proteins. For example, analysis with the bioinformatics platform BOMP (Berven *et al.*, 2004), suggests that 139 β-barrel membrane proteins are encoded in the genome of *Escherichia coli*, and a similar number in the genomes of the deltaproteobacterium *Bdellovibrio* and the epsilonproteobacterium *Helicobacter pylori*. The *Alphaproteobacteria* provide exceptions to this rule, with some species apparently coding for very few β-barrel proteins; only 31 are predicted from the genome of *Brucella mellitensis* and only 17 in the genome of *Rickettsia prowazekii*.

A large proportion of the bacterial species within the alphaproteobacterial group are intracellular symbionts or pathogens (Batut *et al.*, 2004). *Rickettsia* live in the cytoplasm of human host cells and rely on their host for a range of nutrients that, due to redundancy and gene loss, the bacteria are no longer capable of making. As it has been discussed previously (Andersson & Kurland, 1998; Ogata *et al.*, 2001; Sallstrom & Andersson, 2005), *Alphaproteobacteria* that live as symbionts and parasites tend to dispense with genes that are not essential for core functions. Studying their outer membrane protein assembly pathway offers insight into general features of the process. It is possible that, with such a narrow set of substrate proteins, some nonessential components of the assembly pathway have become superfluous. Given that mitochondria from eukaryotic cells share ancestry with *Alphaproteobacteria*, studies on the import and assembly of β-barrel proteins into intracellular bacteria and detailed analysis of the assembly of β-barrel proteins into mitochondrial outer membranes promise further insight (Pfanner *et al.*, 2004; Paschen *et al.*, 2005; Dolezal *et al.*, 2006; Bolender *et al.*, 2008). An intriguing recent report showed *Rickettsia* assembles host-derived voltage-dependent anion channel (VDAC) into its outer membrane – perhaps making up for a shortfall in its own membrane protein genes by ‘importing’β-barrel proteins from its host's cytoplasm (Emelyanov & Vyssokikh, 2006).

## The export and assembly of outer membrane proteins in *E. coli*

**Fig. 2:**
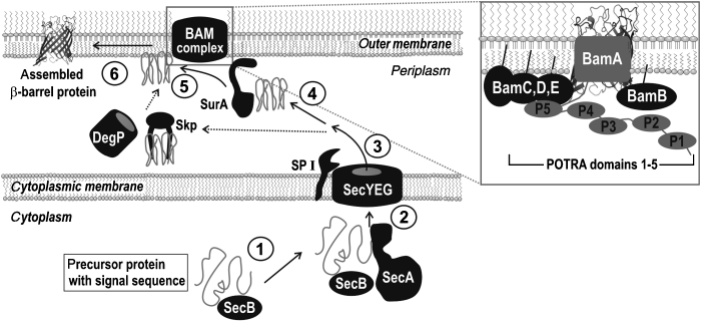
In *Escherichia coli*, outer membrane proteins are synthesized in the cytoplasm as precursors with a signal sequence where they are recognized by the chaperones SecB and SecA (1). SecA assists translocation through the SecYEG complex in the inner membrane (2), and the signal sequence is processed by SP I (3). The substrate proteins are assisted across the periplasm by the chaperone SurA (4), and delivered to the BAM complex (5), to catalyse insertion into the outer membrane (6). Other chaperones, Skp and DegP, might cooperate to help ensure transfer to the outer membrane; DegP can also function as a protease to degrade misfolded outer membrane proteins in situations of environmental stress (Young & Hartl, 2003; Krojer *et al.*, 2008).

Our understanding of β-barrel protein assembly into the outer membrane of *E. coli* is summarized in Fig. 2. Classic studies by Beckwith and Silhavy defined many of the components of the protein secretion pathway (Bassford *et al.*, 1991; Tamm *et al.*, 2004; Mogensen & Otzen, 2005; Ruiz *et al.*, 2006) and recent work by many labs, as reviewed in the following pages, is revealing the mechanisms driving protein secretion and membrane protein assembly.

The β-barrel outer membrane proteins are made as precursors with an N-terminal leader sequence, and these precursor proteins interact with the cytoplasmic factors SecB and SecA, in a process that can also be facilitated by the cytoplasmic chaperone DnaK (Qi *et al.*, 2002; Scott & Barnett, 2006; Chen *et al.*, 2007; Driessen & Nouwen, 2007; Papanikou *et al.*, 2007). After translocation through the SecYEG complex in the inner membrane, the leader sequence is processed by the SP-I signal peptidase and the unfolded protein then associates with the chaperone SurA (Tamm *et al.*, 2004; Mogensen & Otzen, 2005). A second chaperone, Skp, can bind these substrate proteins but perhaps only if they fail to productively interact with SurA (Bitto & McKay, 2003; Kleinschmidt, 2003; Walton & Sousa, 2004; Sklar *et al.*, 2007a). SurA transfers partially folded β-barrels to an ‘insertion and assembly complex’ in the outer membrane. In *E. coli*, the core subunit of this complex is YaeT, a member of the Omp85 protein family, and it is associated tightly with four lipoproteins called YfgL, YfiO, NlpB and SmpA (Wu *et al.*, 2005; Ruiz *et al.*, 2006). This complex, recently dubbed the β-barrel assembly machinery complex (forBAM; Misra, 2007), completes the insertion and assembly of β-barrel protein substrates.

To determine whether equivalent components are found ubiquitously and particularly in the *Alphaproteobacteria*, we made use of hidden Markov models (HMMs) developed to describe each of the known components of the β-barrel assembly pathway. Table 1 summarizes the results for the intracellular pathogens *R. prowazekii* and *Brucella melitensis* and the free-living species *Caulobacter crescentus*. For more broad comparison, we included in the analysis a betaproteobacterium *Neisseria meningitidis*; two seminal studies on outer membrane assembly in *N. meningitidis* first identified Omp85 (Genevrois *et al.*, 2003; Voulhoux *et al.*, 2003). The process of β-barrel protein assembly is highly conserved with Omp85 family members found in all bacteria that have outer membranes (Gentle *et al.*, 2004; Cavalier-Smith, 2006), and many of the other components of the pathway are also highly conserved. However, several distinctions are apparent, as discussed in the following sections.

**Table 1:** HMMs for each protein family were used to find components of the outer membrane assembly pathway in all bacteria for which complete genome information is available

Component*	Bacterial species†				
	
	*Caulobacter*	*Rickettsia*	*Brucella*	*Escherichia*	*Neisseria*
SecB	Q9A224	Q9ZE76	Q8YE23	P0AG86	Q9JY16
SecA	P38380	Q9ZCX7	Q8YJG2	P10408	Q9JYK8
SecY	Q9A8T3	Q9ZCS5	Q8YHM0	P0AGA2	Q7DDS8
SecE	Q9A3J8	P50054	Q8YHQ3	P0AG96	Q9K1J4
SecG	Q9A7K4	Q9ZE68	Q8YHF4	P0AG99	Q7DD68
SPase I	Q9A806	Q9ZE32	Q8YG73	P00803	Q9K056
SPase II	Q9AAA6	Q9ZDC4	Q8YES8	P00804	P65265
Skp	Q9A712	Q9ZDR1	Q8YB18	P0AEU7	Q9K1H1
SurA	Q9A7N3	O05951	Q8YG95	P21202	Q9K186
Omp85	Q9A711	Q9ZE03	Q8YHH0	P0A940	Q9K1H0
YfgL	Q9A7R7	Q9ZDU2	None	P77774	None
YfiO	Q9A6U9	Q9ZDY1	Q8YI58	P0AC02	Q9K0B1
NlpB	None	None	None	P0A903	Q9JZR5
SmpA	Q9A8I8	Q9ZCG9	Q8YGH5	P0A938	Q9K1F0

* Building of HMM models and database search were performed with the hmmer package version 2.3.2. (Eddy, 1998), and used to search the uniprot data set (Release 12.4, containing swiss-prot Release 54.4 and trembl Release 37.4) as described previously for mitochondrial protein translocase components (Dolezal *et al.*, 2006).

† The species listed in the table are *Caulobacter cresentus* CB15, *Rickettsia prowazekii, Brucella melitensis* 16M, *Escherichia coli* K12 and *Neisseria meningitidis* serogroup B.

## Cytoplasmic chaperones SecA and SecB

SecB is a molecular chaperone that is involved in binding signal sequences of various outer membrane protein precursors as they emerge from the ribosome (Lecker *et al.*, 1990; de Cock *et al.*, 1992; Ernst *et al.*, 1994; Behrmann *et al.*, 1998; Baars *et al.*, 2006; Ureta *et al.*, 2007). It then shuttles these proteins to the SecA motor at the SecYEG translocon found in the inner membrane. The interaction between the two chaperones results in the transfer of the preproteins to SecA and the release of SecB (Fekkes *et al.*, 1997). SecB was suggested to be present only in *Proteobacteria* (Driessen, 2001; Scott & Barnett, 2006), and this exclusive distribution is confirmed in the HMM search. SecB is highly conserved across the five classes of *Proteobacteria*, including *Alphaproteobacteria*. In the crystal structure of SecB the protein is a dimer of dimers, and in solution the tetrameric form is in dynamic equilibrium with the dimeric form and this equilibrium is important for substrate transfer to SecA (Topping *et al.*, 2001; Dekker *et al.*, 2003; Randall *et al.*, 2005). A serine residue (Ser^22^) that sits at the dimer interface is phosphorylated in the SecB protein of *E. coli* (Macek *et al.*, 2008). The functional consequence of phosphorylation is unknown, but the Ser^22^ residue is conserved in the alphaproteobacterial (and other) SecB sequences.

SecA is a multidomain ATPase, which binds to nascent outer membrane proteins after their synthesis in the cytoplasm, assisting their translocation through the SecYEG translocon in the cytoplasmic membrane (Driessen & Nouwen, 2007; Gelis *et al.*, 2007; Papanikou *et al.*, 2007; Rapoport, 2007). In *Alphaproteobacteria*, SecA has a conserved structure with all the domains known for the *E. coli* homologue: the core DEAD (or helicase) motor, the C-domain and preprotein-binding domain (PBD), which confer the specificity on the DEAD motor and presumably enables protein insertion into the SecYEG complex in the inner membrane.

## SecYEG translocon

Most of the bacterial proteins which cross the cytoplasmic membrane are translocated through a heterotrimeric SecYEG complex (Breyton *et al.*, 2002; van den Berg *et al.*, 2004; Smith *et al.*, 2005; Driessen & Nouwen, 2007; Rapoport, 2007). Biochemical and genetic experiments (Bieker *et al.*, 1990; Driessen *et al.*, 1991) together with structures of bacterial (*E. coli*; Breyton *et al.*, 2002) and archaeal (*Methanoccocus janaschii*; Van den Berg *et al.*, 2004) SecYEG complexes revealed that the membrane pore is formed solely by the 10 transmembrane segments of the SecY subunit, with the smaller SecE and SecG subunits on the periphery. SecE forms an external clamp for the helical bundle of SecY, while the proposed role of the nonessential SecG is to stabilize the complex and stimulate cytosolic SecA activity (Papanikou *et al.*, 2007; Sugai *et al.*, 2007). In each species of *Alphaproteobacteria* analysed, the SecYEG complex is predicted to be equivalent to that found in other bacteria: SecY predicts to have 10 transmembrane helices with both the N- and C-termini facing the cytoplasm, SecE has a single predicted transmembrane segment, as found in most bacteria, with the exception of the *Gammaproteobacteria* (e.g. *E. coli*) and some *Betaproteobacteria*. SecG would span the cytoplasmic membrane twice, as is expected to be typical for all bacteria.

## The signal peptidase

Several signal peptidases have been implicated in aspects of protein secretion, and the signal sequences that dictate β-barrel protein export across the inner membrane are removed by the leader peptidase SP I (signal peptidase I). The structure and function of the leader peptidase in *E. coli* has been studied and shown to consist of a protease domain, oriented in the periplasm, which is anchored to the cytoplasmic membrane by two transmembrane α-helices (Dalbey *et al.*, 1997). The same topology is predicted for the leader peptidase from the betaproteobacterium *N. meningitidis* and from other *Proteobacteria*.

Bioinformatic protein sequence analysis tools, TMpred and DAS, both suggest that the leader peptidase from *Alphaproteobacteria* each have a single predicted transmembrane domain, but otherwise conform to the domain structure expected for leader peptidases (Paetzel *et al.*, 2002). Leader peptidase is considered the ancestor of the mitochondrial IMP complex that consists of two related proteases Imp1 and Imp2 (Gakh *et al.*, 2002). While most *Alphaproteobacteria* have a single peptidase similar to Imp1 (Burri *et al.*, 2005), in *C. crescentus* there are two genes encoding leader peptidases where one, CC1559, has the sequence signatures characteristic of the mitochondrial Imp2, rather than Imp1. It is through a similar evolutionary process of gene duplication and specialization, that the Imp2 protease of mitochondria is thought to have been derived (Burri *et al.*, 2005).

## The periplasmic chaperones Skp and SurA

Work with *E. coli* mutants shows SurA to be the major chaperone for delivery of proteins across the periplasm to the outer membrane (Tamm *et al.*, 2004; Hennecke *et al.*, 2005; Ruiz *et al.*, 2006; Sklar *et al.*, 2007a). At least two other periplasmic chaperones, Skp and DegP, are involved in outer membrane assembly (Tamm *et al.*, 2004; Mogensen & Otzen, 2005), but have been suggested to serve as ‘back-up’ to assist proteins that fail to interact productively with SurA (Sklar *et al.*, 2007a). DegP appears to assist folding for a subset of outer membrane proteins; *degP*-null strains show decreased levels of the major outer membrane proteins OmpA, OmpF and, to a lesser extent, OmpC. This chaperone assembles into large oligomers of 6, 12 or 24 copies, with the 12- and 24-mer sequestering folded outer membrane protein protomers, but not oligomers, prior to their insertion and assembly in the outer membrane (Krojer *et al.*, 2008). DegP can also play a degradative role, being directly responsible for proteolysis of ‘terminally misfolded’ outer membrane proteins (Young & Hartl, 2003).

Members of the Skp protein family are present in diverse bacteria, including all proteobacterial groups and other bacteria that have outer membranes including *Treponema pallidum, Bacteroides thetaiotaomicron, Chlamydophila caviae* and *Deinococcus radiodurans* (Alcock *et al.*, 2008). The cyanobacteria have an outer membrane and Omp85 (Bölter *et al.*, 1998; Gentle *et al.*, 2004; Schleiff & Soll, 2005), but their genomes do not encode a Skp homologue (Alcock *et al.*, 2008).

SurA sequences have been identified in many groups of bacteria, such as the *Chlorobiaceae* and groups typified by *D. radiodurans* and *Cytophaga hutchinsonii* and are highly conserved suggesting that this chaperone, like Skp, was derived early in evolution. However, at least two genera of *Alphaproteobacteria* (*Anaplasma* and *Ehrlichia*) have no sequence that matches the characteristic features of SurA. It is likely then that a secondary loss of SurA has occurred in these groups, reflecting the common trend of gene loss that occurs in intracellular *Alphaproteobacteria* (Batut *et al.*, 2004; Sallstrom & Andersson, 2005). In the absence of SurA, either the Skp and DegP chaperones that are present in *Anaplasma* and *Ehrlichia* perform the essential function provided by SurA in other organisms or, perhaps, a novel chaperone might work in the place of SurA.

In *E. coli* and other *Gammaproteobacteria*, SurA is composed from four domains: an N-terminal domain, two PPIase domains and a C-terminal domain (Behrens, 2002; Bitto & McKay, 2003). Curiously, the SurA sequences found in *Alphaproteobacteria* have either one or no PPIase domains (Alcock *et al.*, 2008), demonstrating that these domains are not fundamental to SurA function. While the PPIase domains are characteristic of SurA from *E. coli*, a mutant SurA protein lacking both PPIase domains still binds β-barrel substrates effectively (Behrens *et al.*, 2001; Hennecke *et al.*, 2005).

## Omp85

Members of the Omp85 family of proteins are found in all groups of bacteria with outer membranes, and in mitochondria and chloroplasts which were derived from intracellular bacteria in the course of evolution (Gentle *et al.*, 2004, 2005; Schleiff & Soll, 2005; Cavalier-Smith, 2006; Bos *et al.*, 2007). Omp85 family members had been identified as vaccine candidates in several groups of bacterial pathogens (reviewed by Gentle *et al.*, 2005) when pioneering work in *N. meningitidis* showed Omp85 to be essential for outer membrane biogenesis (Genevrois *et al.*, 2003; Voulhoux *et al.*, 2003).

Omp85 proteins are defined by the occurrence of two domains: an N-terminal region containing multiple polypeptide-transport-associated (POTRA) domains and a C-terminal β-barrel domain. The recent structure of a related protein, FhaC, provides a reasonable model for the barrel domain of Omp85 (Clantin *et al.*, 2007). In *E. coli* and in *Neisseria*, deletion of any one of the POTRA domains has at least partial effects on outer membrane protein assembly (Robert *et al.*, 2006; Kim *et al.*, 2007). Recently, the three-dimensional structure of the POTRA domains from the Omp85 protein YaeT were solved and provide the basis for a model for how Omp85 can bind diverse peptide sequences, enabling it to handle its numerous β-barrel protein substrates (Kim *et al.*, 2007; Misra, 2007). The alphaproteobacterial homologs of Omp85 all appear to have five POTRA domains.

In *E. coli*, the Omp85 protein YaeT is in a complex with four lipoproteins: YfgL, NlpB, YfiO and SmpA (Stenberg *et al.*, 2005; Wu *et al.*, 2005; Malinverni *et al.*, 2006; Sklar *et al.*, 2007b). These have recently been renamed BamA(YaeT), BamB(YfgL), BamC(NlpB), BamD(YfiO) and BamE(SmpA) to reflect their functional interactions in the BAM complex (Misra, 2007). Analysis of each of the POTRA domain deletion mutants suggests that while the first POTRA domain is not required for interactions with the lipoprotein partners, POTRA domains 2, 3 and 4 are required to mediate interactions with BamB. In addition, POTRA domain 3 has been suggested to mediate augmentation of β-strand formation in substrate proteins. The fifth POTRA domain is crucial for interactions with the other partner lipoproteins (Kim *et al.*, 2007) and, in *Neisseria meningitidis*, a truncated form of Omp85 lacking the first four POTRA domains is sufficient for outer membrane biogenesis (Robert *et al.*, 2006; Bos *et al.*, 2007).

## Lipoprotein partners of Omp85

**Fig. 3:**
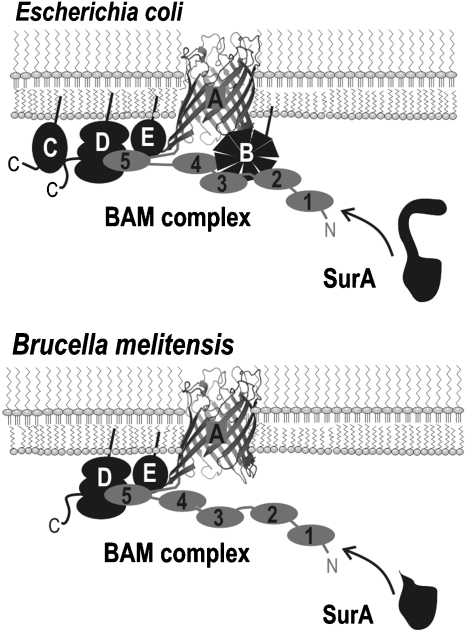
The BAM complexes in *Escherichia coli* and *Brucella melitensis*. The interactions between subunits of the BAM complex in *E. coli* are depicted. The POTRA domains are labelled numerically 1–5: POTRA domains 2, 3 and 4 are required to mediate interactions with YfgL (BamB=‘B’), while the fifth POTRA domain is crucial for interactions with YfiO (‘D’) and SmpA (‘E’), with BamD serving as the docking point for NlpB (‘C’) (Malinverni *et al.*, 2006; Kim *et al.*, 2007; Sklar *et al.*, 2007b). In *Brucella melitensis*, the SurA chaperone is diminished lacking the PPIase domains, and BamB (YfgL) and BamC (NlpB) are lacking from the BAM complex.

Outer membrane lipoproteins are synthesized as precursor proteins in the cytoplasm, with a characteristic N-terminal signal sequence followed by a key cysteine residue. The precursor protein is translocated into the periplasm by the Sec protein machinery in the inner membrane, and processed by signal peptidase II (Table 1) and modified by a lipoprotein diacylglyceryl transferase (Narita *et al.*, 2004; Tokuda & Matsuyama, 2004). The lipid moiety anchors the lipoprotein in the inner or outer leaflet of the outer membrane: leaving a large proportion of the lipoprotein available for interaction with other outer membrane proteins. Genetic analysis identified the four lipoproteins BamB, BamC, BamD and BamE (YfgL, YfiO, NlpB and SmpA, respectively) that interact with Omp85 (Fig. 3), and coprecipitation and other analyses (Wu *et al.*, 2005; Malinverni *et al.*, 2006; Sklar *et al.*, 2007b) suggest a basis for the overall architecture of the BAM complex: (1) there is direct interaction between BamA and BamC (Malinverni *et al.*, 2006), (2) there is a direct interaction between BamA and BamB (Malinverni *et al.*, 2006), (3) binding of BamD occurs through interactions it makes with the C-terminus of BamC (Malinverni *et al.*, 2006; Vuong *et al.*, 2008) and (4) BamE interacts directly with BamA, BamC and BamD, but not with BamB (Sklar *et al.*, 2007b).

**Fig. 4:**
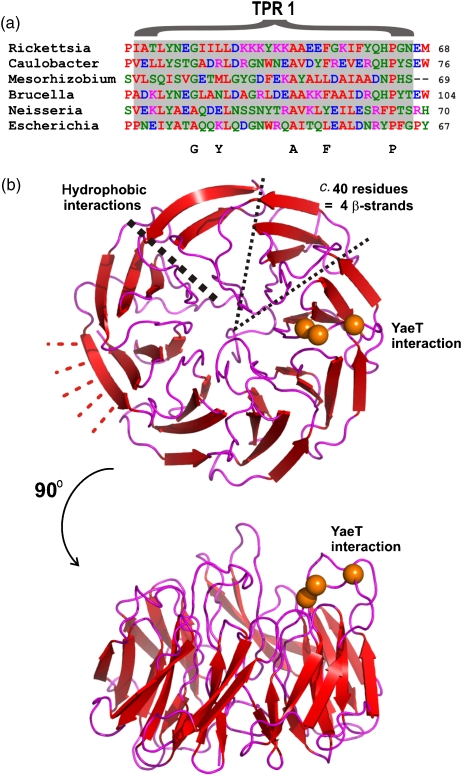
Sequence analyses of the *Alphaproteobacteria* suggest BamD (YfiO) has at least five TPR motifs and BamB (YfgL) could have a β-propeller structure. (a) BamD (YfiO) sequences from *Alphaproteobacteria* were analysed with three independent TPR prediction strategies (SMART, http://smart.embl-heidelberg.de/; HHpred, http://toolkit.tuebingen.mpg.de/hhpred; and TPRpred, http://toolkit.tuebingen.mpg.de/tprpred), revealing the presence of five TPR motifs in various *Alphaproteobacteria*, including *Rickettsia, Caulobacter* and *Mesorhizobium*. The TPR is a degenerate motif with very few strictly conserved positions – the position of the consensus residues G–Y–A–F–P are shown. Although TPR signatures are not clear in all BamD sequences (such as the one from *Escherichia coli*), clustalw aligns the BamD homologs readily and at least some of the key residues found in TPR motifs are evident in all species. The alignment corresponding to the first TPR motif (‘TPR1’) is shown. (b) BamB (YfgL) sequences from *Alphaproteobacteria* were analysed with SMART to determine the presence of seven or eight β-propeller motifs. Homology searching using HHpred suggests BamB from *E. coli* is most similar to proteins with an eight-bladed β-propeller fold. Each of the blades would interact via hydrophobic contacts (heavy black line) and the outer β-strand can make additional hydrogen bond contacts (red lines). BamB from *E. coli* can readily be modelled using six of the β-propeller structures in the Protein Data Bank (2AD6, 1YIQ, 1KB0, 1W6S, 1FLG and 1KV9) as template structures. The model structure of BamB is shown from two views. In this structural model the three mutations in BamB (L173, L175, and R176; the Cα atoms of each are highlighted as orange spheres), that cause defects for docking to the POTRA domains of BamA (Vuong *et al.*, 2008), come together in one of the β-propeller motifs.

The gene encoding BamD is essential for viability of *E. coli* (Onufryk *et al.*, 2005; Wu *et al.*, 2005; Malinverni *et al.*, 2006) and the BamD lipoprotein is found ubiquitously in Gram-negative bacteria (Malinverni *et al.*, 2006; Table 1). This includes non-*Proteobacteria* such as *Treponema, Chlorobium* and *Chlamydophila*. In *N. gonorrhoeae* the homologous protein is a peptidoglycan-associated lipoprotein called competence lipoprotein (ComL) and, as a consequence, BamD homologs are often annotated as encoding a ‘DNA uptake lipoprotein’; a transposon insertion into the middle of ComL resulted in reduced cell size, aberrant cellular morphology and transformation deficiency (Fussenegger *et al.*, 1996), presumably a result of the altered outer membrane properties of these mutants. The BamD protein in *Rickettsia* and other *Alphaproteobacteria* each strongly predict to have at least three tetratricopeptide repeat (TPR) motifs, but comparisons of all these sequences in multiple sequence alignments suggest each protein probably contains six TPR helix-turn-helix structures. The consensus sequence for the TPRs is not stringent, and is not well conserved in the BamD from *E. coli*, however, overall sequence similarities would suggest a homologous TPR-rich structure is present in all BamD proteins (Fig. 4a). TPRs are structural elements that enable protein–protein interactions and have been found operating in a number of protein transport pathways (Blatch & Lassle, 1999; D'Andrea & Regan, 2003). For example, the mitochondrial protein import receptor Tom70 is built from multiple TPR elements (Chan *et al.*, 2006; Wu & Sha, 2006) and binds β-barrel substrate proteins *en route* to the mitochondrial equivalent of the BAM complex (Chan *et al.*, 2006). A TPR-rich structure might enable BamD to bind partner proteins (like BamC) and/or substrate proteins.

*Escherichia coli* mutants lacking BamC have mild defects in outer membrane biogenesis (Onufryk *et al.*, 2005; Wu *et al.*, 2005; Malinverni *et al.*, 2006; Sklar *et al.*, 2007b. A BamC homolog has not been found in any of the *Alphaproteobacteria* (Table 1), a distinct (or highly diverged) protein might fulfil the function of BamC. Alternatively, if the function of BamC is redundant another component of the BAM complex might compensate in these organisms.

Mutants in which the *yfgL* gene, encoding BamB, has been deleted have reduced levels of outer membrane proteins (Onufryk *et al.*, 2005; Ruiz *et al.*, 2005). The BamB protein from *Alphaproteobacteria* has seven or eight predicted Pyrrolo-quinoline quinone (PQQ) enzyme repeats, a motif representative of β-propeller structures (Jawad & Paoli, 2002) found in some enzymes and in protein domains involved in protein-protein interactions. Homology searches suggest that both the alphaproteobacterial and the *E. coli* BamB fit the profile of domains with an eight-bladed β-propeller fold (Fig. 4b). The PQQ enzyme active site residues are not present in BamB, indicating this protein is unlikely to be involved in enzymatic catalysis. If BamB does have a β-propeller structure, it could interact with β-strands in the POTRA of BamA and/or assist in stabilizing nascent β-strands in substrate proteins. While BamB is found in several species of *Alphaproteobacteria* including *Caulobacter* and *Rickettsia*, no related sequence was found in *Brucella*. BamB is also absent from the Omp85 complex in *Neisseria* (Table 1; Bos *et al.*, 2007). It was recently shown that cargo-bound SurA can bypass interaction with BamB and interact directly with BamA (Vuong *et al.*, 2008). It may be that only some protein substrates interact with BamB prior to assembly.

*Escherichia coli* mutants lacking BamE have defects in outer membrane protein assembly (Sklar *et al.*, 2007b). This protein is found ubiquitously in *Proteobacteria*, though no related proteins were found in other groups of bacteria with outer membranes. The structure of the homologue, called OmlA, from *Xanthomonas* has been solved (Vanini *et al.*, 2008). As in *E. coli*, OmlA in *Pseudomonas* and *Xanthomonas* is required for outer membrane integrity (Ochsner *et al.*, 1999; Sklar *et al.*, 2007b; Fuangthong *et al.*, 2008) *omlA* mutants show increased susceptibility to antibiotics (such as rifampin and chloramphenicol) and detergents. Given their high degree of sequence similarity, the structure of OmlA will be important for interpreting interactions BamE makes with other components of the BAM complex.

## How do β-barrels assemble?

Understanding the folding pathway for any membrane protein is a considerable challenge. For β-barrel assembly, it is now possible to start to reconcile biophysical analyses that have looked at folding reactions undergone by purified proteins, with our growing appreciation of the components mediating the assembly pathway *in vivo*. A β-barrel can be considered a continuous β-sheet, wrapped to a cylinder. The assembly of a β-barrel, therefore, consists of three processes:

1. preventing the somewhat hydrophobic polypeptide from misfolding before its localization within a membrane environment,
2. folding of β-strands into the β-sheet, with the first and final strand interactions completing the cylindrical shape and
3. insertion of the barrel into the lipid phase of the outer membrane.

Biophysical evidence suggests the substrate proteins would be in a partially structured form in the periplasm (reviewed by Tamm *et al.*, 2004) and that periplasmic chaperones such as SurA are needed to maintain the ‘periplasmic intermediate’ (Lazar & Kolter, 1996; Bulieris *et al.*, 2003; Hennecke *et al.*, 2005). *In vitro* models of outer membrane protein insertion predict a so called ‘molten disc’ intermediate at the bilayer interface, with β-strands sitting flat on the membrane (Kleinschmidt & Tamm, 2002; Tamm *et al.*, 2004). SurA is, therefore, the prime candidate to assist in process (1).

The β-strands in outer membrane proteins have a sequence Φ–X–Φ (where Φ represents an aromatic residue) at least in the C-terminal strand, and mutations in this motif impair assembly of outer membrane proteins (Struyve *et al.*, 1991; de Cock *et al.*, 1997; Robert *et al.*, 2006). SurA has been shown to bind directly to the C-terminal Φ–X–Φ motif (Bitto & McKay, 2003), as has Omp85 (de Cock *et al.*, 1997; Robert *et al.*, 2006; Bos *et al.*, 2007). The POTRA domains of Omp85/BamA provide a means to receive substrates from chaperones like SurA. The POTRA domains have been suggested to facilitate templating of β-strands in the nascent outer membrane protein substrate and, if the lipoprotein BamB has a β-propeller structure, it too might facilitate strand formation in this way to assist in the completion of process (2). If its role were simply to assist the function of SurA and Omp85, this redundancy of function would explain why BamB is absent in some bacteria and why corresponding *yfgL* mutants of *E. coli* show only relatively minor defects in outer membrane assembly.

The third process is perhaps the one we understand least about. What mechanism is used to insert proteins into the hydrophobic core of the outer membrane? The β-barrel domain of Omp85/BamA might increase the kinetics of strand insertion by providing some local distortions in the lipid population, assisting intermediate forms of a barrel to gradually assemble and enter the membrane (3). Alternatively, the proteinaceous environment created by the components of the BAM complex in the periplasm might favour a barrel forming, to enable a more dramatic *en bloc* insertion of the barrel into the plane of the outer membrane.

One class of substrate protein, the autotransporters, is providing an excellent means to interrogate the mechanism of Omp85/BamA function. Autotransporters consist of an N-terminal ‘passenger domain’ and a C-terminal β-barrel domain (Henderson *et al.*, 2004; Jacob-Dubuisson *et al.*, 2004; Newman & Stathopoulos, 2004; Dautin & Bernstein, 2007). The secretion of the passenger domain requires the insertion of the β-barrel into the outer membrane and the translocation of the passenger domain across the outer membrane. But do these two reactions occur one after the other, as suggested by the name ‘autotransporter’? When secretion is complete, what remains of the fragment connecting the two domains can be found within the lumen of the barrel (Oomen *et al.*, 2004; Meng *et al.*, 2006; Barnard *et al.*, 2007), and this ‘vapour trail’ suggests the passenger passes through the barrel pore to cross the outer membrane. However, recent work identified a novel intermediate in the periplasm whose topology resembles that of the protein after passenger domain translocation, suggesting the fragment connecting the two domains was placed inside the barrel lumen before insertion into the outer membrane (Ieva *et al.*, 2008). It represents a thermodynamically impressive feat if the BAM complex is capable of inserting into the outer membrane an autotransporter with the huge passenger domain prepositioned on its outer surface (Jain & Goldberg, 2007; Ieva *et al.*, 2008). It should be noted, whichever mechanism is used to translocate autotransporter passengers across the outer membrane, the β-barrel has a limited tolerance for folded elements in the passenger domain (Jong *et al.*, 2007).

## What mitochondria tell us about outer membrane assembly

Mitochondria are found in all eukaryotic cells, having been derived from intracellular bacterial symbionts (Yang *et al.*, 1985; Gray *et al.*, 1999). There has been debate over whether hydrogenosomes and mitosomes found, in place of ‘classic’ mitochondria, in some unicellular eukaryotes are metabolically specialized mitochondria. However it is now clear that these organelles all share a common ancestry, with a common set of protein translocases that were developed in mitochondria for the import of proteins from the cytosol (Dolezal *et al.*, 2006). In the course of evolution most of the genes encoding mitochondrial proteins, including those coding for β-barrel outer membrane proteins, were transferred to the nucleus (Lang *et al.*, 1999; Andersson *et al.*, 2003). The number of β-barrel proteins in the outer mitochondrial membrane is probably small (estimates in yeast are *c*. 15), though the major porin VDAC accounts for around 25% of the protein mass of the membrane (Burri *et al.*, 2006; Zahedi *et al.*, 2006; Hoogenboom *et al.*, 2007; Young *et al.*, 2007). The outer membrane of mitochondria is a phospholipid bilayer, with phosphatidylcholine, phosphatidylethanolamine and phosphatidylinositol the major lipid constituents (de Kroon *et al.*, 1997, 1999).

**Fig. 5:**
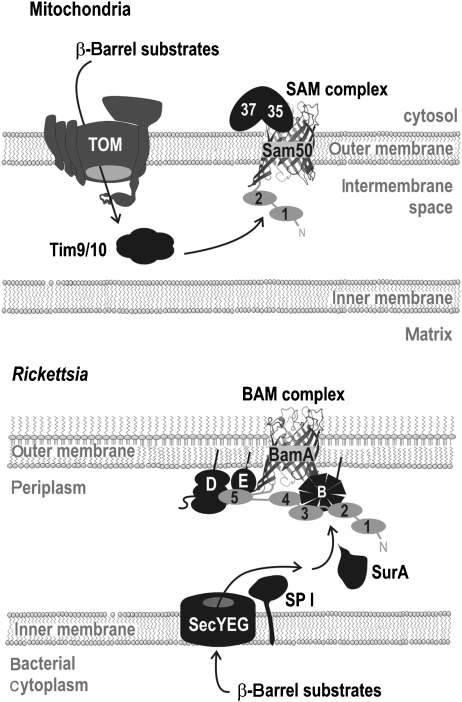
Assembly pathways for β-barrel proteins in mitochondria and *Alphaproteobacteria*. In eukaryotes, β-barrel proteins are translated in the cytosol, transported across the mitochondrial outer membrane by the TOM complex and passed to the inner surface of the SAM complex for assembly. Passage through the intermembrane space depends on tiny TIM chaperones, such as the Tim9/10 complex (Pfanner *et al.*, 2004; Paschen *et al.*, 2005; Bolender *et al.*, 2008). The final steps of assembly depend on the metaxins, Sam35 and Sam37 (‘35’ and ‘37’), on the outer face of the membrane (Pfanner *et al.*, 2004; Paschen *et al.*, 2005; Bolender *et al.*, 2008; Chan & Lithgow, 2008). The *Rickettsia* are considered the closest living relatives to the progenitor of mitochondria, and use a BAM complex mechanism largely equivalent to that found in other bacteria.

Despite being imported from the cytosol, mitochondrial β-barrel proteins are still inserted into the outer membrane from the inner surface (Fig. 5). This requires that proteins be recognized and translocated through the outer membrane by the protein translocase in the outer mitochondrial membrane (TOM complex). The TOM complex has no counterpart in bacteria. In the intermembrane space, topologically equivalent to the bacterial periplasm, β-barrel proteins are chaperoned by tiny TIM chaperones (Fig. 5). While the tiny TIM proteins show some structural similarities to Skp chaperones (Webb *et al.*, 2006), and functional similarities to SurA (Alcock *et al.*, 2008), there is no obvious ancestry to relate the mitochondrial and bacterial chaperones. The assembly of β-barrel proteins into the mitochondrial outer membrane is driven by the sorting and assembly machinery (SAM complex) and at the core of the SAM complex is a protein called Sam50, a member of the Omp85 protein family (Kozjak *et al.*, 2003; Paschen *et al.*, 2003; Gentle *et al.*, 2004). Reflecting their ancestry, mitochondrial Sam50 proteins are most closely related to the Omp85 proteins from *Alphaproteobacteria* (Gentle *et al.*, 2004).

The mitochondrial SAM complex receives substrate proteins from the tiny TIM chaperones, and in no eukaryotes have homologs of SurA or Skp been found (Alcock *et al.*, 2008). This demonstrates that Omp85 proteins do not need to contact a SurA chaperone to productively receive substrate proteins. The hidden Markov model searches reported here find no evidence for BamB, BamC, BamD or BamE homologs in eukaryotes. In a sense then, mitochondria can be considered as representing an end-point to the gene loss seen in *Rickettsia* and *Brucella* in regards to components of the outer membrane assembly machinery.

The absence of the four lipoproteins and the SurA chaperone in mitochondria has an important corollary, with the mitochondrial protein Sam50 predicted to have only 1–2 POTRA domains. These POTRA domains are not essential for Sam50 to function, with the metaxin Sam35 serving as the ‘receptor’ for β-barrel substrates to bind the SAM complex and initiate assembly (Chan & Lithgow, 2008; Kutik *et al.*, 2008). It is impossible to tell whether, in the course of evolution, the loss of the POTRA domains was causative for loss of the genes encoding SurA and the lipoproteins, or whether it happened subsequently. However, the findings in the mitochondrial system provide independent support to the proposition that the five POTRA domains in the bacterial Omp85 are important because they are needed to organize the lipoprotein partners and the SurA chaperone.

## Concluding remarks

The assembly of β-barrel proteins into membranes is an essential process in Gram-negative bacteria, mitochondria and plastids. The significance of the process is underscored by observations that even relatively small perturbations to the assembly process, such as mutations in nonessential components of the BAM complex, compromises the barrier function of the outer membrane leaving mutant bacteria hypersensitive to antibiotics. Our understanding of the mechanism of β-barrel assembly is benefiting from comparative approaches, with findings on the assembly pathway in organelles informing studies on the pathway in bacteria, and *vice versa*.

With the availability of vast genome sequence data from so many species of bacteria it has become possible to make comparative sequence analyses, to add further value to the work carried out with model bacteria such as *E. coli* and *N. meningitidis*. Some protein features, the presence of multiple TPR motifs in BamD and of multiple β-propeller signatures in BamB, are not readily apparent in an analysis of a single polypeptide. However, these structural features become strikingly apparent when a set of homologous proteins from across bacterial species is analysed as a group.

This is an exciting time for the study of membrane proteins. An increase, slow but sure, in the number of structures solved for membrane proteins has increased our appreciation of how polypeptides can be organized in biological membranes. Improved assays for tracking the assembly of membrane proteins in distinct systems provides a means to cross-fertilize our knowledge of any given system. A prime example of this is how the discovery of Omp85 in bacterial outer membranes led to the characterization of the SAM complex in the outer membrane of mitochondria, and the further understanding of the ‘BAM complex’ in bacteria. Three clear questions now present themselves, with answers that would largely complete our understanding of β-barrel membrane protein assembly.

Firstly, where and how does folding of a polypeptide into a β-barrel occur? The barrels may fold into the lipid environment of the outer membrane, but it seems increasingly likely that folding occurs before the encounter with the bilayer. But would this be in the periplasm *per se*, assisted by soluble chaperones such as SurA, or in the grasp of the various periplasmically exposed components of the BAM complex?

Secondly, how do the external loops of β-barrels fold? These loops are crucial for the function of β-barrel membrane proteins often forming the occlusions that provide selectivity to the β-barrel pores. In mitochondria, there are external factors, the metaxins, bound to the SAM complex that might assist these folding reactions (Pfanner *et al.*, 2004; Paschen *et al.*, 2005; Dolezal *et al.*, 2006; Chan & Lithgow, 2008), but we know of no specific factors bound to the outer surface of the bacterial BAM complex.

Thirdly, can Omp85 do more than just assemble β-barrels? While it is clear Omp85 plays a direct role in outer membrane protein insertion (Voulhoux *et al.*, 2003; Doerrler & Raetz, 2005), other studies have shown that cells in which the expression of Omp85 has been shut down accumulate lipopolysaccharide and phospholipids in their inner membranes, likely *en route* to the outer membrane. This defect could be a secondary effect of improper outer membrane protein assembly, but it remains possible that Omp85 also aids lipid insertion via a similar mechanism to protein insertion: through creating a ‘disturbance’ in the outer membrane that could accelerate the transfer of substrates into the hydrophobic core.

Addressing these questions promises a more complete picture of the assembly of bacterial outer membranes.
